# Effect of peripheral arterial disease on the onset of lactate threshold during cardiopulmonary exercise test: study protocol

**DOI:** 10.1136/bmjopen-2016-012763

**Published:** 2016-12-19

**Authors:** Angela Key, Tamara Ali, Paul Walker, Nick Duffy, Mo Barkat, Jayne Snellgrove, Francesco Torella

**Affiliations:** 1 Department of Respiratory Medicine, University Hospital Aintree, Liverpool, UK; 2 Faculty of Health and Life Sciences, University of Liverpool, Liverpool, UK; 3 Liverpool Vascular and Endovascular Service, University Hospital Aintree, Liverpool, UK; 4 Faculty of Science and Engineering, University of Liverpool, Liverpool, UK

**Keywords:** OXYGEN DELIVERY, PREOPERATIVE ASSESSMENT, VASCULAR SURGERY, REHABILITATION MEDICINE

## Abstract

**Introduction:**

Cardiopulmonary exercise test (CPET) is widely used in preoperative assessment and cardiopulmonary rehabilitation. The effect of peripheral arterial disease (PAD) on oxygen delivery (VO_2_) measured by CPET is not known. The aim of this study was to investigate the effect of PAD on VO_2_ measurements during CPET.

**Methods and analysis:**

We designed a prospective cohort study, which will recruit 30 patients with PAD, who will undergo CPET before and after treatment of iliofemoral occlusive arterial disease. The main outcome measure is the difference in VO_2_ at the lactate threshold (LT) between the 2 CPETs. The secondary outcome measure is the relationship between change in VO_2_ at the LT and peak exercise pretreatment and post-treatment and haemodynamic measures of PAD improvement (ankle–brachial index differential). For VO_2_ changes, only simple paired bivariate comparisons, not multivariate analyses, are planned, due to the small sample size. The correlation between ABI and VO_2_ rise will be tested by linear regression.

**Ethics and dissemination:**

The study was approved by the North West-Lancaster Research and Ethics committee (reference 15/NW/0801). Results will be disseminated through scientific journal and scientific conference presentation. Completion of recruitment is expected by the end of 2016, and submission for publication by March 2017.

**Trial registration number:**

NCT02657278.

**Strengths and limitations of this study**

- First study to address the influence of peripheral arterial disease (PAD) on cardiopulmonary exercise test (CPET) results.
- Prospective design, established CPET protocol.
- Small sample size.
- Inclusion of patients with proximal PAD only, treated with multiple interventional modalities.

## Introduction

Cardiopulmonary exercise testing (CPET) is frequently used in the preoperative assessment of elderly patients,^1^ as well as in the evaluation and follow-up of patients with cardiorespiratory disease.^2^ Peripheral arterial disease (PAD) is highly prevalent in this population, due to its age and the presence of cardiovascular risk factors.^3^ CPET documents cardiorespiratory fitness by various means, including the measurement of systemic oxygen delivery (VO_2_) at the lactate threshold (LT—the moment, during exercise, when muscles start working anaerobically) and at peak exercise. Low values suggest poor fitness and may indicate that surgery is of inappropriately high risk. Patients with PAD may develop ischaemia during leg exercise not because of poor cardiorespiratory reserve, but, independent of cardiorespiratory performance, because the blood supply to the muscles is impaired, resulting in early lactate release. On this basis, the LT may not reflect cardiorespiratory status, risk and prognosis in this group of patients. In addition, screening for PAD prior to CPET is not currently advocated or practiced.

To the best of our knowledge, there is no literature documenting or quantifying the effect of PAD on the results of CPET. Our hypothesis is that correction of PAD may cause improvement in VO_2_ proportional to the degree of improvement in the peripheral circulation. The aim of this study is to determine whether VO_2_ during CPET is influenced by the presence of haemodynamically significant PAD. More specifically, our research question was: in patients with PAD, does improvement in blood flow to the leg muscles result in a rise in VO_2_ at LT and peak exercise, as measured by CPET?

## Methods

### Design

In order to answer our research question, we designed a prospective cohort study recruiting patients scheduled to undergo percutaneous or surgical correction of proximal (iliofemoral) occlusive PAD. We chose patients with iliofemoral (rather than infrainguinal) disease because of the greater muscle mass experiencing ischaemia during CPET in this population (quadriceps, glutei). Inclusion and exclusion criteria are summarised in table 1. We excluded patients with critical ischaemia because this condition (severe pain at rest with/without tissue loss) might affect their ability to perform a CPET.

**Table 1:** Entry criteria

Inclusion	Exclusion
Ability and willingness to give written informed consent	Critical ischaemia as presenting symptom (rest pain and/or tissue loss)
Iliofemoral PAD scheduled for surgical or percutaneous treatment	Age <18 years
Ability to perform a CPET on a cycle ergometer	Previous amputation
Intermittent claudication as presenting symptom	Inability to perform a CPET on a cycle ergometer
Age ≥18 years	Uncontrolled hypertension
	Unstable angina
	Acute coronary syndrome within 6 weeks of the test
	Terminal illness
	Advanced cancer
	Psychiatric illness or dementia precluding informed consent

### Recruitment

Patients are recruited among those referred to the Liverpool Vascular and Endovascular Service for treatment of their PAD. Potential candidates are approached at the time of clinic attendance to determine interest in the study and offered a patient information leaflet as well as verbal information. Alternatively, they receive a study letter containing the patient information leaflet by post. In either case, patients are then approached by the study team by telephone >48 hours later to confirm participation. All participants are asked to provide written informed consent, which is obtained by either the first or the senior author.

### Intervention

Patients undergo symptom limited CPET before and 4 weeks after surgical or endovascular correction of their PAD. The CPET protocol is described in detail in previous publications from our group.^4^ ^5^ The test is performed according to the American Thoracic Society/American College of Chest Physicians recommendations.^6^ ^7^ It includes ECG monitoring, measurement of ventilator parameters and recording of Borg breathlessness and leg fatigue score every minute. At the end of the test, the reason for cessation is documented. The test is incremental with a 10–25 W ramp aiming for the participant to exercise for 10–12 min based on predicted peak workload. The ramp chosen is one which would be expected to result in 8–10 min loaded exercise based on predicted maximum workload and ventilation. The same increment will be used for the pretreatment and post-treatment test. The test involves 3 min rest, 2 min free-pedal followed by continuous ramping until volition. Recording continues for 5 min recovery. The tests are blindly reported by two experienced clinicians (ND and PW). In event of disagreement, consensus is achieved between the two reporters by discussion. Other recorded variables include age, gender, height, weight, BMI, smoking status, haemoglobin concentration, ankle–brachial index (ABI—measured before and after treatment of PAD), medications and comorbidity. Resting flow-volume loops are used to derive forced expiratory volume in 1 s and forced vital capacity. Ventilation and gas exchange variables derived by CPET include VO_2_, absolute and weight-adjusted, ventilatory equivalents for oxygen and carbon dioxide (VE/VO_2_; VE/VCO_2_), oxygen pulse (VO_2_/heart rate), work rate and heart rate; all measured at estimated LT and at peak exercise.

### Primary outcome measure

The difference in VO_2_ at LT between the two CPETs.

### Secondary outcome measures

The relationship between change in VO_2_ at LT and peak exercise pretreatment and post-treatment and haemodynamic measures of PAD improvement (ABI differential).

### Sample size

We could not find, in the literature, any data on patients with PAD undergoing CPET. We thus arbitrarily assumed that a difference in VO_2_ at LT of 1 (SD 1.5) mL/kg/min would be deemed clinically significant, and calculated that 26 patients would be required to demonstrate this difference, at a 5% significance level and with 90% power. We thus decided to recruit 30 patients, in order to allow for a drop-out rate of 10–15%. This sample size will be achievable within 1 year, considering the patient throughput of the Liverpool Vascular and Endovascular Service.

### Study outline

**Figure 1:**
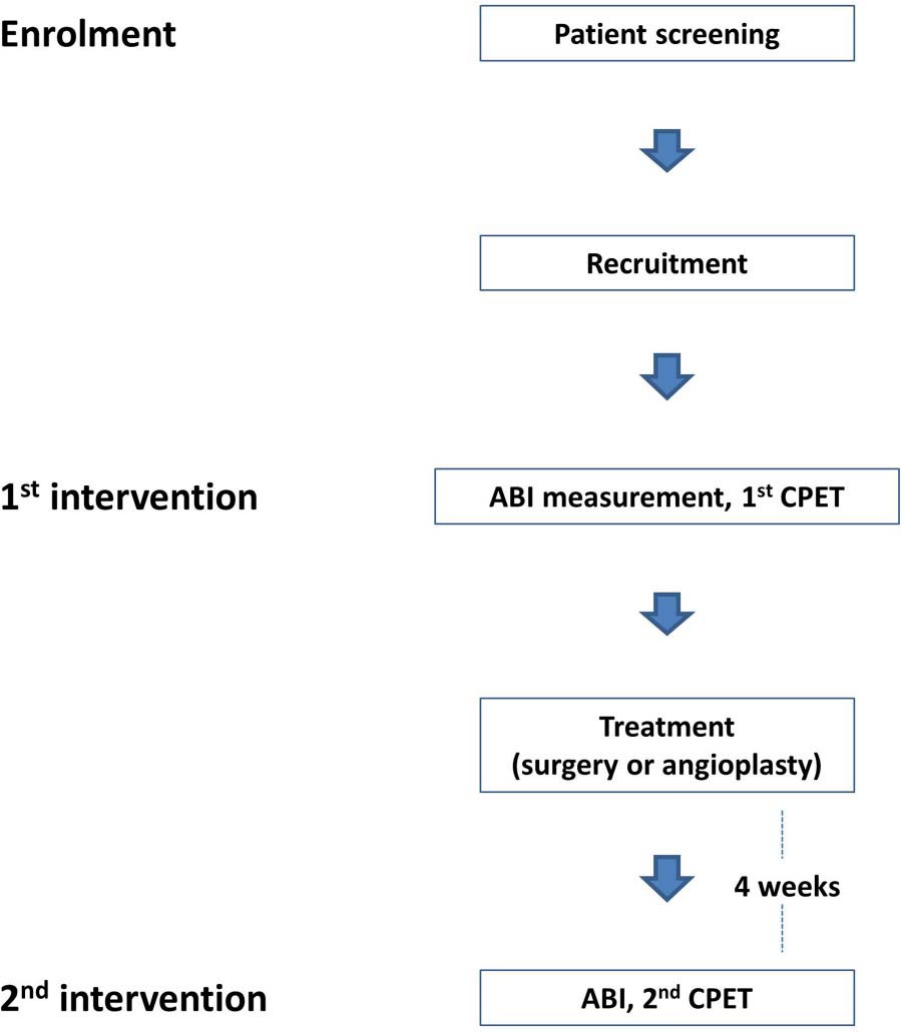
Study outline.

See figure 1. Recruitment is ongoing, with expected completion at the end of 2016. The study was registered at http://www.clinicaltrials.gov (NCT02657278) on 13 January 2016.

### Data analysis

Data are stored in a password-protected hospital server, accessible only by the researchers, according to internal information governance rules. Data will be analysed on completion of the study. We do not plan to perform any interim analyses. Continuous variables will be presented with mean and SD if normally distributed, or median and IQR if not. Parametric or non-parametric paired tests will be used according to the underlying distributions. For VO_2_, only simple paired bivariate comparisons, not multivariate analyses, are planned, due to the small sample size. The correlation between ABI and VO_2_ rise will be tested by linear regression (log transformation of data will be performed, if necessary, prior to this analysis).

### Ethics and governance

The conduct of the study is monitored by the sponsor (University Hospital Aintree Research and Development, Lower Lane, Liverpool, UK), which also provides indemnity. Screening, recruitment and adverse event logs are updated in real time, as necessary, by the research team. Adverse events are immediately reported to the sponsor, as appropriate. Owing to the short duration of the study and the low likelihood of adverse events from the intervention, formal interim safety assessments are not planned.

## Discussion

This study was designed to ascertain whether PAD influences the results of CPET, by seeking an improvement in VO_2_ in patients treated for PAD, and by correlating this improvement (if any exists) with ABI differential measurements. The study was conceived because of lack of information, in the literature, on the effect of PAD on systemic VO_2_ measured by CPET by cycle ergometry, a commonly performed test, in different settings, to evaluate cardiorespiratory fitness. The study was not designed to provide conclusive results, rather as an exploratory investigation. Any evidence of a positive effect of (correction of) PAD on the outcome measures may stimulate further research and inform future sample size estimates. Furthermore, assuming a positive finding, the study may suggest caution in interpreting the results of CPET in patients with PAD, pending further evidence. It may also induce clinicians to screen for PAD prior to CPET. Although one of the secondary outcome measures is the correlation between changes in VO_2_ and ABI, the study is not specifically powered for this, nor could it be, due to the lack of evidence in the literature. Furthermore, ABI is only a crude measurement of the effect of treatment of PAD, and not solely dependent on the presence of PAD, and it is influenced by infrainguinal disease, whose presence is not addressed by the treatment of the patients included in this study.

In conclusion, this study will provide further insight on the use and interpretation of CPET in the elderly, and evaluate PAD as a potential limiting factor of cardiorespiratory performance in this group of patients.
